# Aging with Down Syndrome—Where Are We Now and Where Are We Going?

**DOI:** 10.3390/jcm10204687

**Published:** 2021-10-13

**Authors:** Melissa J. Alldred, Alessandra C. Martini, David Patterson, James Hendrix, Ann-Charlotte Granholm

**Affiliations:** 1 Nathan Kline Institute, NYU Grossman Medical School, 140 Old Orangeburg Rd, Orangeburg, NY 10962, USA; Melissa.Alldred@nki.rfmh.org; 2 Department of Pathology and Lab. Medicine, University of California Irvine, Irvine, CA 92697, USA; ac.martini@uci.edu; 3 Knoebel Institute for Healthy Aging, University of Denver, Denver, CO 80208, USA; David.Patterson@du.edu; 4 LuMind IDSC Foundation, 20 Mall Road, Suite 200, Burlington, MA 01801, USA; jhendrix@lumindidsc.org; 5 Department of Neurosurgery, CU Anschutz, 12631 East 17th Avenue, Aurora, CO 80045, USA

**Keywords:** Down syndrome, aging, biomarkers, neuropathology, Alzheimer’s disease

## Abstract

Down syndrome (DS) is a form of accelerated aging, and people with DS are highly prone to aging-related conditions that include vascular and neurological disorders. Due to the overexpression of several genes on Chromosome 21, for example genes encoding amyloid precursor protein (*APP*), superoxide dismutase (*SOD*), and some of the interferon receptors, those with DS exhibit significant accumulation of amyloid, phospho-tau, oxidative stress, neuronal loss, and neuroinflammation in the brain as they age. In this review, we will summarize the major strides in this research field that have been made in the last few decades, as well as discuss where we are now, and which research areas are considered essential for the field in the future. We examine the scientific history of DS bridging these milestones in research to current efforts in the field. We extrapolate on comorbidities associated with this phenotype and highlight clinical networks in the USA and Europe pursuing clinical research, concluding with funding efforts and recent recommendations to the NIH regarding DS research.

## 1. Introduction

### 1.1. Background 

**Figure 1:**
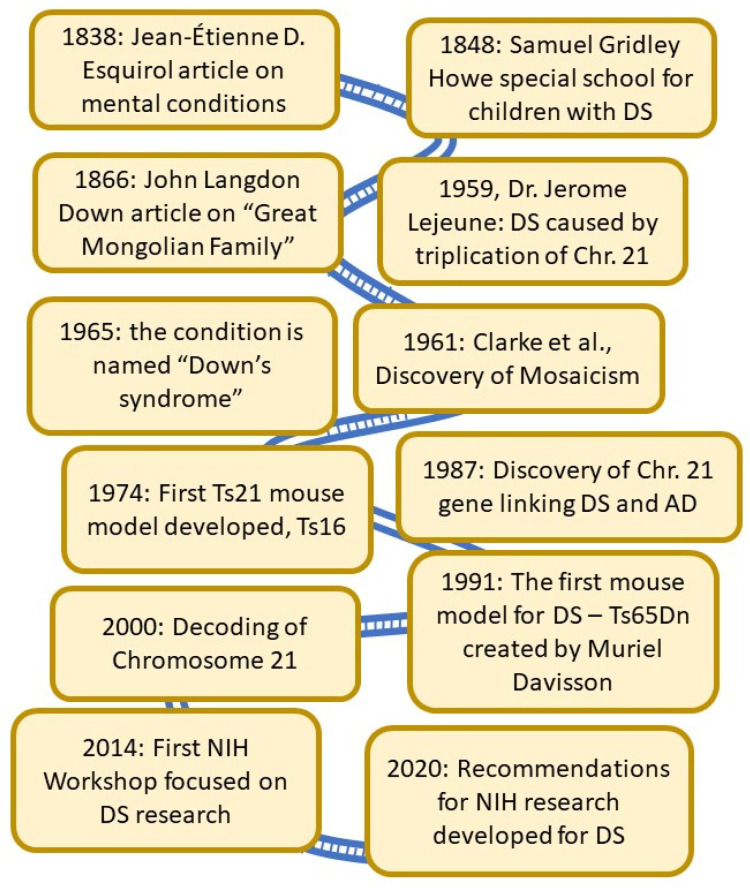
A Brief History of DS research. As noted in this outline, the first known publications focused on children with DS occurred in the mid-1800s. In 1866, Dr. John Langdon Down wrote about “the Great Mongolian Family”, and almost a century later the condition was named after him. Dr. Patterson and his colleagues finished decoding Chr. 21 in the late 1990s, and the sequence was first published in year 2000—only the second chromosome to be fully decoded. Thirty years ago, the first non-lethal mouse model for DS—Ts65Dn—was created by Dr. Muriel Davisson. This model has been used extensively to detect the connection between aging-related brain degeneration and DS and is still used today.

Down Syndrome (DS) is caused by a complete or segmental triplication of human chromosome 21 (HSA21) [1] and is the most frequent genetic cause of intellectual disability (ID) [2]. People with DS present with developmental abnormalities [3], systemic alterations in the peripheral system, as well as neurological and cognitive deficits (Table 1) [2,4,5,6]. The most common causes for emergency room (ER) visits in children with DS are ear, nose, and throat issues [7]. In addition, muscle dystonia [8], and thyroid disorders are common in both children and adults with DS [9,10,11]. Further, during mid-life people with DS develop pathological changes associated with Alzheimer’s Disease (AD), including plaques, neurofibrillary tangles (NFTs), degeneration of cholinergic basal forebrain neurons (BFCNs) and other discrete neuronal populations, widespread neuroinflammation, epileptic seizures [12], and endosomal/lysosomal abnormalities [13,14,15,16,17,18,19,20]. Along with AD neuropathology follows progressive cognitive impairment towards dementia [21,22,23,24,25]. Descriptions of clinical symptoms in individuals with DS have been published since the early 1800s (Figure 1).

A publication by Dr. Jean-Étienne D. Esquirol in 1838 on mental conditions included a description of persons with DS [29]. Dr. Edouard Seguin, who studied psychiatry under Dr. Esquirol, went on to publish a book on the physiological method of teaching individuals with DS [30], believing DS was caused by weakness in the nervous system to be treated with motor and sensory training. This early concept has later been proven to be true—cognitive and physical training do impact and improve the activities of daily living (ADL) of person with DS [31,32]. He later worked with Dr. Samuel Gridley Howe [33] in developing training schools. In the United States, Dr. Howe pioneered the establishment of an experimental school in 1848 with the goal of preparing children with mental disabilities to live in society, where innovative and novel alternative methods were used for teaching those with IDs [34,35]. A seminal paper was published by Dr. John Langdon Down in 1866 in which he described a subset of persons with intellectual disabilities which he called the “great Mongolian family”, based on their observed craniofacial abnormalities [36,37]. He found that “*The improvement which training exerts in them is greatly in excess of what would be predicted if one did not know the characteristics of the type. The life expectancy, however, is far below the average*”. He further described deficits in skin elasticity, speech impairments, deficits in coordination, circulation and intellectual disability [36,37]. Later, in 1965, this classification would take on his name in respect for his lifelong care and commitment to those with DS [38]. In 1959, Dr. Jerome Lejeune discovered that DS was caused by the triplication of human chromosome 21, and he received the J.P. Kennedy Foundation research award in 1962 for this discovery [1]. In the early 60s, researchers discovered the presence of so-called mosaicism—a condition where not all cells in the body have trisomy of Chromosome 21 [39]. It also became apparent that segmental trisomy of HSA21 could also cause DS. Study of these partial trisomy cases was important in the genetic characterization and eventual sequencing of HSA21 (see [40] for a discussion of these developments). A decade later, researchers developed the first mouse model for DS—the Ts16 complete trisomy model, which is lethal (Figure 1) [41,42]. In the end of the 1980s, it was reported for the first time that a gene associated with Alzheimer’s disease (AD) was located on Chromosome 21, and thus could be one of the reasons for the high incidence of AD in those with DS [43]. This discovery has had significant importance for continued treatment of adults with DS. Several more years passed before Dr. Muriel Davisson developed a segmental trisomy mouse model, the Ts65Dn mouse, which survives into adulthood and develops many of the clinical and neuropathological signs of aging in DS observed in humans [44,45,46]. Although this mouse model has some confounds and does not replicate the entire genome that is triplicated in humans with DS, it became an important tool in a significant surge in research on biological mechanisms of DS-related medical conditions [24].

Recent medical advances have made it possible to treat many comorbidities in DS, including heart defects such as atrial fibrillation, congenital heart disease, sleep apnea [10,27], deficits in immune response and leukemia [47,48,49,50]. These advances have therefore resulted in increased life span [5,51] and birth survival rates for persons with DS—currently estimated at approximately 1 in ~700 live births in the US [52,53]. Life expectancy for children with DS increased remarkably the last hundred years. In 1920, the average life expectancy for children with DS was only 9 years old, it was 12 years old in 1946, and today it is close to 60 years of age [24]. This has consequently resulted in more people with DS living with aging-related disorders, including AD pathology [13,14,15,16,17,18,19,20,54] and epilepsy [55,56,57], exemplifying the need for clinical studies and therapeutic treatment for individuals with DS, especially regarding aging deficits.

### 1.2. The Decoding of Chromosome 21

DS research reached a new level by the publication of the decoding of HSA 21 in the spring of 2000 (Figure 1) [58]. Some results had been obtained a few years earlier and were reported by a multinational consortium consisting of scientists in the Human Genome Project (HGP) at an international workshop on Human Chromosome 21 Mapping in 1996 [59]. Chromosome 21 was the second human chromosome to be decoded, following Chromosome 22. This consortium concluded that the long arm of chromosome 21 represents approximately 1% of the human genome and described several structural features of the chromosome including the locations of numerous HSA21 breakpoints found in naturally occurring chromosome translocations and experimentally induced rearrangements of the chromosome, the distribution of repeat sequences, and variations in gene density along the chromosome. There were only 3 gaps in the sequence of the q (long) arm of the chromosome. Sequencing of the p (short) arm of the chromosome was much less complete, but in the context of DS this is not a major issue since the short arm of the chromosome does not appear to be relevant to the phenotype of the syndrome. Chromosome 21 was already known to be involved with several conditions including DS, AD, Usher syndrome and Lou Gehrig’s disease before the sequencing was completed [4,58,60,61,62,63,64]. The scientists involved in the Human Genome Project (HGP), provided all data immediately at no cost and without restrictions to all scientists in industry and academia [58]. The greatest impact of mapping HSA21 was no doubt the follow-up research which led to identifying specific genes responsible for traits and comorbidities in DS—work that is not yet completed and has remained in the focus of DS research even today.

### 1.3. Common Age-Related Morbidities in DS

As mentioned previously, people with DS are prone to multiple conditions during the aging process that may shorten life span and affect quality of life [23]. These are outlined in Table 1, along with common developmental co-morbidities of DS [26,65]. From early adulthood, individuals with DS may present with multiple conditions simultaneously associated with progressive aging. The cardiovascular and immune systems appear to be most affected as well as the brain, prompting some investigators to suggest that DS may represent a segmental form of accelerated aging [66]. It is not always clear who is responsible for continued care of persons with DS as they enter adulthood, nor have standards been implemented to ensure that they receive adequate care for all conditions, with the result that these individuals may be lost between pediatrics, general medicine, and geriatrics as their symptoms of aging occur much earlier than in the general population. Included in these debilitating conditions are for example visual and hearing loss, epilepsy, depression, and dementia. Exacerbating this problem, there are not enough experts in health care who can identify and treat aging symptoms in those with DS. Of the comorbidities described in Table 1, dementia appears to be the most common cause of morbidity and mortality in older adults with DS [22,67]. However, dementia is difficult to diagnose in those with DS, who may already have cognitive changes resulting from developmental challenges. Specialized cognitive and other behavioral batteries are needed and are currently being developed in the different clinical networks—hopefully leading to harmonized protocols across the US and other countries. Despite these challenges, many people with DS now survive into their 60s-70s and live healthy and active lives [68].

## 2. Clinical Networks in the US and in Europe

**Figure 2:**
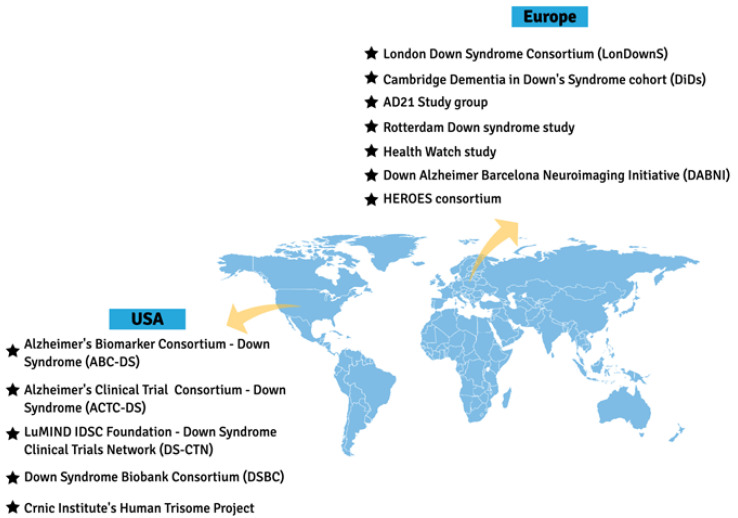
Clinical networks focused on DS in the US and Europe. There are several well-characterized cohorts of adults with DS in Europe, now representing the Horizon 21 consortium, which currently includes 10 different countries in Europe. In the US, there are also several networks, including the AD Biomarker consortium (ABC-DS), the Down syndrome Biobank consortium for collection of brain tissues (DSBC), the LIFE-DSR trial-ready population, and the Crnic Institute’s Human Trisome Project, focused on genetic alterations.

Major advances in the understanding of DS-AD were made over the past decade thanks to the relentless effort of research groups in many countries. Studies generated by these researchers have demonstrated critical points of synergy between DS-AD, early onset AD (EOAD) and late onset AD (LOAD), but also pointed out specific alterations that are characteristic to DS-related AD, and these advances continue to expand through specific clinical networks formed worldwide (Figure 2). Through the combination of two independent groups, the Alzheimer’s Biomarker Consortium-Down Syndrome (ABC-DS) was formed in 2015 and developed into a multi-center, longitudinal study involving multiple performance sites—recently funded as a large multi-site U-grant from the National Institutes of Health (NIH). The largest U.S.-based initiative to target AD in people with DS, it includes analysis of clinical, cognitive, blood and CSF biomarkers, neuroimaging and neuropathology, as well as genetic modifiers. With harmonized protocols and expanding collaborations, ABC-DS will provide critical understanding of DS-AD and enable future clinical trials in this population [64]. This clinical network currently has 11 sites, all located in the US. Launched in 2018, the Alzheimer’s Clinical Trial Consortium—Down Syndrome (ACTC-DS) is a platform to develop and conduct clinical trials targeting treatment and prevention of dementia in the DS population. It aims to create a cohort of adults with DS who are available to participate in clinical trials focused on the discovery of new treatments. This initiative, conducted across 15 sites spanning the US and Europe, will allow researchers to quickly enroll participants in new trials. The Trial Ready-Cohort Down Syndrome (TRC-DS) is the first project to be conducted by the consortium and it will enroll 120 participants in preparation for a phase II clinical trial of an anti-amyloid approach [69]. The LuMIND IDSC Foundation—Down Syndrome Clinical Trials Network (DS-CTN) put together a group of clinical trials sites with the goal of including people with DS in clinical trials and advance effective treatments for this population. Launched in 2018, it currently encompasses 14 sites across 10 states in the US.

In Europe, research groups are also collaborating to obtain clinical, biomarker, and genetic data to enable trials targeted at prevention or treatment of AD in DS. The Horizon21 DS Consortium is formed by 10 European centers, including cohorts from London (The London Down Syndrome Consortium, LonDownS) and Cambridge (Cambridge Dementia in Down’s Syndrome cohort, DiDS) in the UK; Germany (AD21 Study group); Netherlands (the Rotterdam Down syndrome study and the Health Watch study); Spain (Down Alzheimer Barcelona Neuroimaging Initiative, DABNI), France (TriAL21 for Lejeune Institute), and recently included cohorts from Ireland, Norway, and Sweden (see also URL: https://www.horizon-21.org, accessed on 9 April 2021). The focus is to obtain essential clinical data on the progression of AD in DS, including in its early stages, as well as to develop a clinical trial-ready network [67].

Other important initiatives include the Down Syndrome Biobank Consortium (DSBC), that puts together universities in US and Europe at 10 different sites to standardize protocols for collection and procurement of DS brain tissue, the HEROES consortium, and the Crnic Institute’s Human Trisome Project. These important collaborations will help to further advance clinical trials and therapeutic intervention developments that are needed for prevention and treatment of dementia in DS and are working with the pharmaceutical industry to increase their awareness and willingness to include those with DS in clinical trials (Figure 2).

## 3. Past, Current, and Future Clinical Trials

Due to the extremely high prevalence of AD in individuals with DS (70–90%) [70], clinical trials within this population provide a unique opportunity to target not only the cognitive impairment in DS but also the early onset of AD pathology. Individuals with DS represent the largest population group with early onset-AD yet remain under-studied and are often not included in prevalence estimates or clinical trials. In the past decade, the number of clinical trials involving individuals with DS increased significantly. However, of the hundreds of clinical trials for AD on Clinicaltrials.gov, fewer than ten interventional AD trials are conducted in the DS population (Clinicaltrials.gov). This problem is further exacerbated by the difficulties in enrollment and broad exclusion criteria for this population. Some barriers are due to well-intentioned laws governing informed consent in persons with intellectual disabilities or overprotective institutional review boards (IRBs) that prevent people with intellectual disabilities from participating in clinical research [71,72]. Due to these caveats in recruitment, most clinicians must use caution when selecting treatments and doses for their DS patients since there is scant clinical data available even for FDA approved treatments [73]. Many of the past clinical trials within the DS population were limited to Phase 2 trials involving small cohorts, which have shown little to no beneficial effects on cognition but demonstrated safety of treatment for individuals with DS. Further, these trials have secondary benefits in identifying knowledge gaps and novel areas of study. A summary of past and current clinical trials in DS is provided in Table 2.

Clinical trials in DS were performed during gestation and in infants including a combination treatment of folinic acid and L-thyroxine [74], as well as folic acid [81] treatments, aimed to improve cognitive development (Table 2). Past studies on adults with DS included interventional treatments with vitamin E [80,82], epigallocatechin-3-gallate (EGCG) [31,83,84], glulisine [85], memantine [75,76,86], basmisanil [87,88,89,90], and rivastigmine [78,79,91] as well as anti-amyloid treatments [92] and treatments to modulate myoinositol [77], all aimed to prevent or slow the progression of cognitive decline and AD pathological symptoms (Table 2). Preventative studies with the acetylcholinesterase inhibitor donepezil hydrochloride, a commonly prescribed AD drug, were undertaken in younger individuals with DS; however, these studies were terminated prior to outcome measurements due to lack of efficacy [93,94,95], with the only completed study recently (20/04/2021) posting results showing little to no efficacy [96]. While significant efforts were made into these treatments, pharmacological treatments achieved little success when evaluated in placebo-controlled trials [97] and no treatment advanced beyond these small phase 2 trials. However, modest beneficial effects were shown through a combination of health and educational measures bolstering mild successes [28,32,98]. More research is needed to understand the potential of these strategies to improve cognition in children and adults with DS [97]. New clinical research will also benefit from an increased understanding of the basic biologic processes at work in childhood cognitive development [99,100], although this is not the focus of the current review. The past barriers, including recruitment difficulties for these studies, highlight the importance of current clinical networks designed to connect patients to trials and longitudinal studies.

As detailed in Section 2 above, there are several remarkable clinical network initiatives that were implemented with the goal of increasing participation of persons with DS in clinical trials [101,102,103]. In addition to these initiatives, several interventional treatments are also underway. These include trials utilizing gonadorelin (GnRH) [104], nicotine [105] and memantine [106] (Table 2), which are anticipated to be completed within the next year, barring delays due to the COVID pandemic. In addition to the drug treatment trials, therapeutic intervention trials include examining changes in physical activity [107,108,109] on cognition and cognitive decline, use of orthotics [110] to help mobilize those with physical disabilities, which may result in reduced obesity and metabolic dysregulation as well as obstructive sleep apnea treatments [111,112], to reduce the elevated apnea-hypopnea index in patients with DS which is postulated to result in improved cognition.

Although legal and ethical barriers still exist to conducting clinical trials in people with DS, with each new trial more is learned, and obstacles are overcome. While most interventional trials in DS are performed with repurposed drugs or approved devices, these trials will build the foundation for future trials on novel devices or novel pharmaceutical interventions. Indeed, several additional studies involving sleep apnea treatments [113,114,115,116] as well as a novel therapeutic, transcranial photo-biomodulation (tPBM) [117,118], along with an expanded trial on the ACI-24 treatment [119] are all trials that are currently slated to begin recruitment. As more is learned about conducting trials in persons with DS, novel therapy trials should become more common leading to better therapeutic options for people with DS. As described elsewhere, individuals with DS are exceptionally vulnerable to COVID-19 infection [120,121,122,123] with increased morbidity and a 10 times higher mortality than age-matched normosomic persons [124,125]. As medications and vaccines become available for the Sars-Cov-2 virus and potentially more dangerous variants, clinical trials can quickly be implemented due to the developing clinical trials networks in Europe and the US and hopefully also in other countries.

## 4. Recommendations for Future Research Support

In 2020, the National Institutes of Health (NIH) announced that they would update their DS research plan and requested input from the research and the non-profit/family advocacy communities. The planning sessions, which included more than 50 investigators and non-profit stakeholders, were organized by The National Down Syndrome Society (NDSS) and the LuMind IDSC Foundation to develop recommendations for the NIH research plan. Eleven different work groups were formed, and their planning sessions took place separately and in parallel during the spring of 2020, during a raging pandemic. The work groups included every stage of life from development to aging and their task was to identify gaps in current research strategies as well as recommendations for new areas that needed further attention via NIH funding. The recommended strategies for adults and older adults with DS are summarized in Table 3, and the full report is available online in a white paper [126]. The white paper calls for an increased participation of people with DS in clinical trials to confirm that new treatments are safe and effective in this population. This issue has taken on new urgency with the recent FDA approval of Aduhelm for AD. Unfortunately, this drug was approved without the inclusion of adults with DS in the clinical trials, so it is difficult to know if the drug is appropriate for DS associated AD. A discussion regarding Aduhelm and its potential use for individuals with DS has erupted recently (see, e.g., https://www.the-ntg.org/aduhelm-information), and future clinical trials could inform physicians and families whether this new drug could potentially help adults with DS.

## 5. Discussion

**Figure 3:**
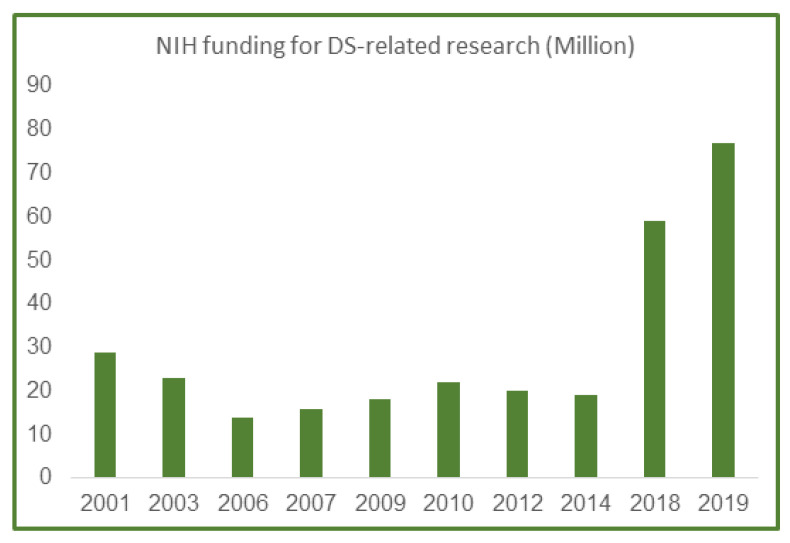
NIH funding in million dollars for DS research from 2001 to 2019. According to the NIH website, total funding for DS research was $29 million in 2001, which has now incrementally increased to more than $70 million in 2019, giving researchers a stellar opportunity to fully investigate clinical comorbidities of DS and AD in the future and with the added guidance provided by the DS NIH plan discussed above.

Based on the increased attention that the DS condition has received the last 10 years in the US and Europe, the funding level for related research also increased incrementally in the US the last several years. In 2001, the National Institutes of Health (NIH) funding for DS research was $29 million out of a $20.5 billion-dollar budget, or 0.0014 percent of the overall budgets (Figure 3). As summarized in Figure 3, funding for DS research decreased yearly until 2018, when a major lift in funding happened due to a couple of targeted NIH-initiated workshops which included experts on all aspects of DS research and spurred new research request for applications (RFAs) in this field.

Despite a very small research budget through 2014 (Figure 3), the NIH recognized the need for additional research on DS to help address the medical and caregiver challenges associated with the increased life expectancy of people with DS during the last few decades and formed the public-private *Down Syndrome Consortium* in 2011. This committee published an updated research plan for Down Syndrome Directions in 2014: *The NIH Research Plan on Down Syndrome* (https://www.nih.gov/include-project/include-project-research-plan).

Importantly, the Fiscal Year 2018 Omnibus Appropriations Report stated the following: “Down syndrome. The agreement directs the NIH Director to develop a new trans-NIH initiative—involving, at a minimum, NICHD, NIA, and NCI—to study trisomy 21, with the aim of yielding scientific discoveries to improve the health and neurodevelopment of individuals with Down syndrome and typical individuals at risk for Alzheimer’s disease…”

With additional funding appropriated for DS research, this directive provided the opportunity to expand research efforts on DS and associated co-morbidities. A new NIH-wide initiative, INvestigating Co-occurring conditions across the Lifespan to Understand Down syndromE (*INCLUDE*), allowed the NIH to build an interdisciplinary research plan across NIH Institutes that has the potential to transform DS clinical care and understanding of biological mechanisms underlying DS-related pathology and clinical care during development, adulthood, and aging. Many researchers have benefitted from the increased funding afforded the last 3 years for DS-related research.

An important area of discovery is biomarkers for early detection of AD—an area that is uniquely suited for application in those with DS since we know early that they will most likely develop AD as they age. Biomarker technology has developed significantly the last few years and now includes not only blood and CSF markers [127,128,129,130], but also exosomal markers [131,132,133], microRNA markers [134,135,136,137], and novel neuroimaging techniques [64]. There are at least 5 microRNAs located on HSA21 [61,138,139,140,141,142,143,144,145]—several which are directly involved in both neuroinflammation and other AD pathology. This field is rapidly emerging and has been recognized as an important area in NIH initiatives and as a tool to determine intervention efficacy during clinical trials.

There is no doubt that continued and expanded support for DS funding will allow major discoveries that can benefit not only those with DS but also the general population suffering from age-related conditions since findings in this field can generally be translated to biological mechanisms that are applicable to all persons with AD. The overall purpose of this review is to highlight age-related neurological conditions in DS, but also to provide novel alternatives for areas to focus on as we move forward in this important research field.

## Figures and Tables

**Table 1:** Common age-associated morbidities in children and adults with DS. These are organized by age and sample citations provided.

Comorbidities in Children and Adults with Down Syndrome		
Comorbidity	Age	References
Infantile spasms	Infant	[26]
Learning, memory and speech problems	Childhood	[3]
Muscle Hypotonia	Childhood	[5,8]
Multiple Organ Anomalies	Childhood	[26]
Congenital heart conditions	Childhood	[10]
Hearing impairment	Children/Adults	[7]
Thyroid disorders	Young/Adult	[9,10,11]
Sleep Apnea	Young adult	[27,28]
Visual impairment	Adult	[23]
Epilepsy	Adult	[12]
Cardiac Valve Disease	Adult	[10]
Dysphasia	Adult	[10]
Dementia	Middle-age	[20,22,25]

**Table 2:** Clinical Trials in the US and Europe. A = Completed, phase 1, no results posted; B = Completed, phase 2, no results posted; C =Terminated, efficacy standard not met; D = Completed, phase 2, results posted, not publicly available; E = Unknown status, no results posted; F = Completed, no phase listed, no results posted.

**Study**	**Clinical Trial # and Results**	**Sponsor**
ACI-24	NCT02738450 ^A^	AC Immune SA
Basmisanil/RG1662	NCT01436955 ^A^; NCT01667367 ^A^; NCT02024789 ^B^; NCT02484703 ^C^	Hoffman/La Roche
Donepezil hydrochloride (Aricept)	NCT00675025 ^C^; NCT00754013 ^C^; NCT00754052 ^C^; NCT00570128	Eisai Inc./Pfizer
epigallocatechin-3-gallate (EGCG)	NCT01394796 ^D^; NCT01699711 [31]	Parc de Salut Mar
Folic Acid	NCT01244347 ^E^	Azienda Ospedaliera Universitaria Integrata Verona
Folinic Acid and L-thyroxine	NCT01576705 [74]	Institut Jerome Lejeune
Glulisine	NCT02432716 ^D^	HealthPartners Institute
Memantine	NCT00240760 ^E^	King’s College, London
Memantine	NCT01112683 [75,76]	University of Colorado, Denver
Memantine	NCT02304302 ^B^	University Hospitals Cleveland Medical Center
Myinositol (ELDN005)	NCT01791725 [77]	OPKO Health, Inc.
Rivastigmine	Clinical Trial # unknown [78]; NCT01084135 [79]	Duke University
Rivastigmine	NCT00748007 ^F^	National Taiwan University Hospital
Sleep Apnea	NCT03267602 ^F^	National Taiwan University Hospital
Sleep Apnea	NCT03942341 ^E^	Fundació Institut de Recerca de l’Hospital de la Santa Creu i Sant Pau
Vitamin E	NCT00056329 [80]; NCT01594346	New York State Institute for Basic Research
**Upcoming/Recruiting Studies**	**Clinical Trial #**	**Sponsor**
Gonadorelin (GnRH)	NCT04390646	Nelly Pitteloud, Centre Hospitalier Universitaire Vaudois
Nicotine	NCT01778946	Vanderbilt University Medical Center
Sleep Apnea	NCT04115878	University of Arizona
ACI-24	NCT04373616	AC Immune SA
Sleep Apnea	NCT04132999	Children’s Hospital of Philadelphia
Sleep Apnea	NCT04198493	Fundació Institut de Recerca de l’Hospital de la Santa Creu i Sant Pau
Sleep Apnea	NCT04801771	Inspire Medical Systems, Inc.
Transcranial Photobiomodulation (tPBM)	NCT04668001	Massachusetts General Hospital
Transcranial Photobiomodulation (tPBM)	NCT04211870	University of Nove de Julho

**Table 3:** Recommendations for future research to the NIH. Eleven groups discussed and finalized a plan presented to the NIH in the spring of 2020 for the suggested focus of future research funding opportunities related to both childhood development and adult medical needs of persons with DS. These are the major points raised by the more than 50 experts involved in the process.

**Recommendations to the NIH Spring 2020**
Define clinical and genetic phenotypes across life course
Expand genetic and epigenetic profiling beyond Chr21
Gather more unbiased -Omics data
Develop and support better DS models (cells, rodents, non-human primate)
Increase interdisciplinary/translational collaborations
Expand support for drug and devise RCT’s across lifespan
Increase life-style studies and interventions including physical fitness, health, and behavior
Develop and disseminate methodology for cognitive/ behavior outcome measures for large, multi-site trials
Expand clinical trial data sharing
Expand support for centralized biorepositories and a single network for DS data across the life span
Support training in clinical research/treatment for DS
Expand inclusion of individuals with DS who have been under-represented and excluded from clinical studies

## Data Availability

This is a review so it does not need this section.
